# IL-6 promotes growth and epithelial-mesenchymal transition of CD133+ cells of non-small cell lung cancer

**DOI:** 10.18632/oncotarget.6570

**Published:** 2015-12-12

**Authors:** Soo Ok Lee, Xiaodong Yang, Shanzhou Duan, Ying Tsai, Laura R. Strojny, Peter Keng, Yuhchyau Chen

**Affiliations:** ^1^ Department of Radiation Oncology, University of Rochester School of Medicine and Dentistry, Rochester, NY 14642, USA

**Keywords:** non-small cell lung cancer, IL-6, CD133+, cancer stem cells, self-renewal

## Abstract

We examined IL-6 effects on growth, epithelial-mesenchymal transition (EMT) process, and metastatic ability of CD133+ and CD133– cell subpopulations isolated from three non-small cell lung cancer (NSCLC) cell lines: A549, H157, and H1299. We developed IL-6 knocked-down and scramble (sc) control cells of A549 and H157 cell lines by lentiviral infection system, isolated CD133+ and CD133– sub-populations, and investigated the IL-6 role in self-renewal/growth of these cells. IL-6 showed either an inhibitory or lack of effect in modulating growth of CD133– cells depending on intracellular IL-6 levels, but there was higher self-renewal ability of IL-6 expressing CD133+ cells than IL-6 knocked down cells, confirming the promoter role of IL-6 in CD133+ cells growth. We then examined tumor growth of xenografts developed from CD133+ cells of A549IL-6si vs. A549sc cell lines. Consistently, there was retarded growth of tumors developed from A549IL-6si, CD133+ cells compared to tumors originating from A549sc, CD133+ cells. The effect of IL-6 in promoting CD133+ self-renewal was due to hedgehog (Hhg) and Erk signaling pathway activation and higher Bcl-2/Bcl-xL expression. We also investigated whether IL-6 regulates the EMT process of CD133− and CD133+ cells differently. Expression of the EMT/metastasis-associated molecules in IL-6 expressing cells was higher than in IL-6 knocked down cells. Together, we demonstrated dual roles of IL-6 in regulating growth of CD133– and CD133+ subpopulations of lung cancer cells and significant regulation of IL-6 on EMT/metastasis increase in CD133+ cells, not in CD133– cells.

## INTRODUCTION

Lung cancer is the predominant cause of cancer deaths in both men and women [1]. While lung cancer is heterogeneous in cell types, it is generally divided into two major subtypes: small cell lung carcinomas (SCLCs) and non-small cell lung carcinomas (NSCLCs), with the latter comprising 85% of all lung cancer cases [2]. Despite decades of research and clinical trials testing different therapeutic interventions, the treatment outcome of lung cancer remains unsatisfactory.

Recent evidence supports the hypothesis that tumors contain putative cancer stem cells (CSCs). The existence of CSCs in lung tumors and in established NSCLC cell lines has been reported [3, 4]. It has been suggested that CSCs are responsible for chemo- [5] and radio-resistance [6, 7], thus research may reveal potential therapeutic targets in improving the outcome of NSCLC treatments.

Interleukin-6 (IL-6) is a cytokine that provokes a broad range of cellular and physiological responses. It is a pleiotropic cytokine that influences antigen-specific immune responses and inflammation [8]. In patient sera of many types of cancers, the IL-6 level is elevated and suggested to be associated with poor clinical outcome [9, 10]. The implication of IL-6 in NSCLC progression has been suggested. Liao et al. [11] showed that a high IL-6 level is associated with shorter overall survival in NSCLC. Pine et al. [12] observed a correlation between patient sera level of IL-6 within two years prior to the subsequent diagnosis of lung cancer. In addition, the circulating IL-6 level has been suggested as a prognostic marker for survival in advanced NSCLC patients treated with chemotherapy [13]. Nevertheless, in analysis of patient sera or cell lines, inconsistent results were obtained. In sera analyses, IL-6 was detectable in 29 of 75 patients with lung cancer (39%) and not detectable in patients with benign lung diseases [14]. In cell line studies, only 53% of NSCLC cell lines express IL-6 mRNA and protein [15]. Bihl et al. [16] suggests existence of two subtypes of NSCLC cells: IL-6-dependent and IL-6-independent.

Meanwhile, recent publications have revealed a significant association of the IL-6 gene promoter polymorphism with NSCLC. The IL-6-174G/C and 174G/G genotypes were suggested as one of the biological markers in the etiology of NSCLC [17, 18]. The IL-6 antibodies have been already used in *in vitro* studies (tocilizumab, [19]), in mouse experiments (siltuximab, [20]), and Phase I clinical studies (clazakizumab [formerly ALD518, BMS-945429]) [21].

Recently, several groups reported the role of IL-6 in promoting CSC growth. Yi et al. [22] showed that the use of IL-6 receptor (IL-6R) led to inhibition of CSC growth, indicating the IL-6 role in promoting CSC growth. Liu et al. [23] reported the IL-6 role in enriching lung CSC-like cells by epigenetic control of p53 and p21 molecules. In contrast, the reports on the effects of IL-6 on modulating total NSCLC cell growth have been controversial. Yamaji et al. [15] and Bihl et al. [16] did not observe any influence of IL-6 on NSCLC cell growth, while Takizawa et al. [24] reported an inhibitory effect of IL-6 on A549 cell growth. However, Kim et al. [19] reported on the promoter role of IL-6 in proliferation of several NSCLC cell lines by showing inhibitory effect of the IL-6 antibody. To clarify this issue, we were determined to investigate the IL-6 role in CD133+, CSC-like and CD133– non-CSC cells separately.

Besides the IL-6 role in regulating the growth of lung cancer cells or CSCs, the IL-6 role in controlling the epithelial-mesenchymal transition (EMT) process has also been suggested [25, 26], and the role of IL-6 in regulating the EMT process in CSCs has never been addressed. Therefore, we conducted studies on the IL-6 effects on regulating the EMT/metastasis of CD133+ and CD133– subpopulation cells.

## RESULTS

### Isolation and characterization of CD133+ cells from NSCLC cell lines

**Figure 1:**
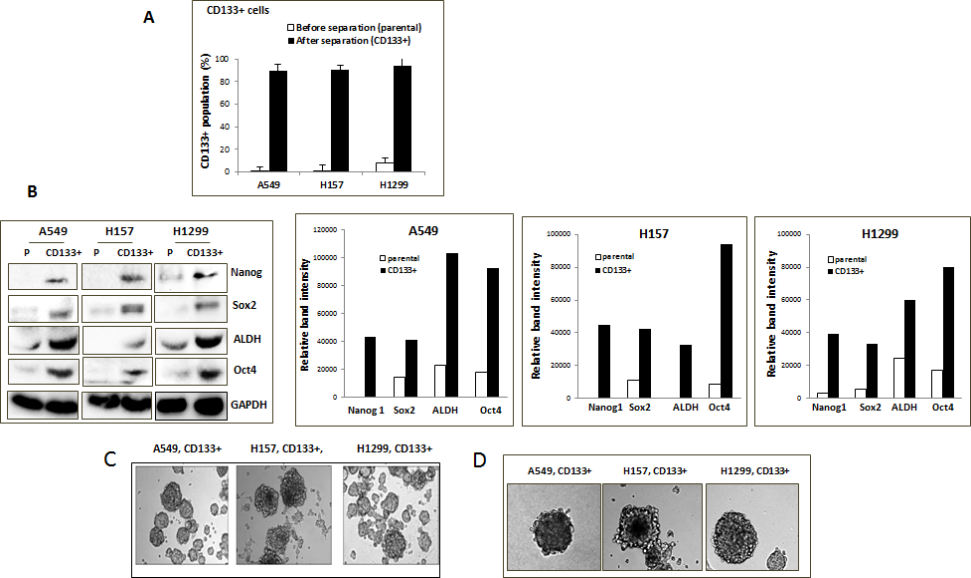
Isolation of CD133+ CSC-like cells (**A**) CD133+ cell population, before and after immunomagnetic separation. CD133+ cells were isolated from A549, H1299, and H157 cells by the immunomagnetic sorting using the CD133+ antibody-conjugated magnetic microbeads. The isolated CD133+ cells were stained by CD133+ antibody and the % of stained population was analyzed by flow cytometry. (**B**) Expression of CSC markers in parental (P) and isolated CD133+ cells of NSCLC cell lines. Cell lysates were obtained from total parental and isolated CD133+ cells, and expressions of the indicated CSC marker proteins in these cells were analyzed by Western blot analyses. Quantitation by densitometry was shown on right. (**C**) Cell growth of CD133+ cells in non-adhered culture condition. (**D**) Sphere formation assay. The cell suspension containing CD133+ cells were mixed with Matrigel (1:1, v/v) and sphere formation assays were performed.

We have isolated CD133+, CSC-like cell population of A549, H1299, and H157 NSCLC cell lines by immunomagnetic separation using the CD133 antibody conjugated-microbeads. The CD133 molecule is the most widely used surface marker for the NSCLC CSC, and previous studies have shown that the CD133+ cells exhibited biological features of CSCs [27, 28]. Flow cytometry analysis has confirmed the purity of the isolated CD133+ cells from the immunomagnetic separation, with greater than 90% positivity of CD133 expression cells (Figure 1A). In all three cell lines, CD133+ cells constituted only a minority of total cells in the parental cell lines, showing varied percentages from 0.8 to 8.2%. The H1299 cell line showed the highest percentage of CD133+ population among the three cell lines. To examine whether the isolated CD133+ cells had CSC characteristics, we analyzed expression of the typical CSC markers Nanog [27, 29], Oct4 [4], Sox2 [27], and ALDH [29] in parental vs. CD133+ NSCLC cells. High expression levels of these CSC markers were consistently detected in isolated CD133+ cells, but not in parental cells (Figure 1B, quantitation shown in right side panels). The CD133+ cells did grow in sphere forms in low-adherence culture conditions in serum-free media supplemented with growth factors (Figure 1C), as well as grow in spheres when mixed with Matrigel (Figure 1D). Such anchorage-independent growth is a known characteristic of CSC [30]. Based on these results, we applied the enriched CD133+ and parental (CD133–) cells as sources of putative CSC and non-CSCs in subsequent experiments.

### Effects of exogenous IL-6 on the growth/self-renewal of CD133– and CD133+ subpopulations of NSCLC cells *in vitro*

**Figure 2:**
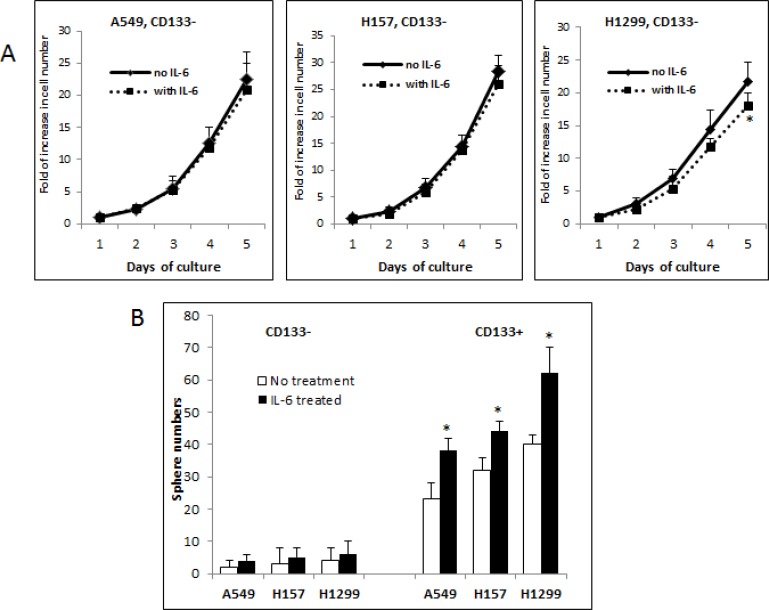
Differential roles of IL-6 in modulating growth/self-renewal of CD133− vs. CD133+ cells of NSCLC cell lines (**A**) Growth analysis of CD133− cells upon exogenous IL-6 treatment. The CD133− cells of A549, H157, and H1299 cell lines were pre-treated with IL-6 (10 ng/ml) (or vehicle as control) for 5 days and cell growth at each day was analyzed by direct cell counting. (**B**) Sphere formation assay. The cell suspension containing CD133− and CD133+ cells were mixed with Matrigel (1:1, v/v) and sphere formation assays were performed. The spheres of larger than 50 μm diameter were counted. Quantification was shown on lower panel (*p < 0.05).

We tested effects of exogenous IL-6 on the growth/self-renewal of the CD133– and CD133+ cells of three NSCLC cell lines. We detected no significant effect on the growth of the CD133– cells in the A549 and H157 cell lines, while observing the reduction in the CD133– cell growth in the H1299 cell line upon IL-6 addition (Figure 2A). In contrast, we found a significant stimulatory effect on the self-renewal of CD133+ cells of all three cell lines in sphere formation assay (Figure 2B).

### Effects of silencing endogenous IL-6 on the growth of CD133– and CD133+ cells

**Figure 3:**
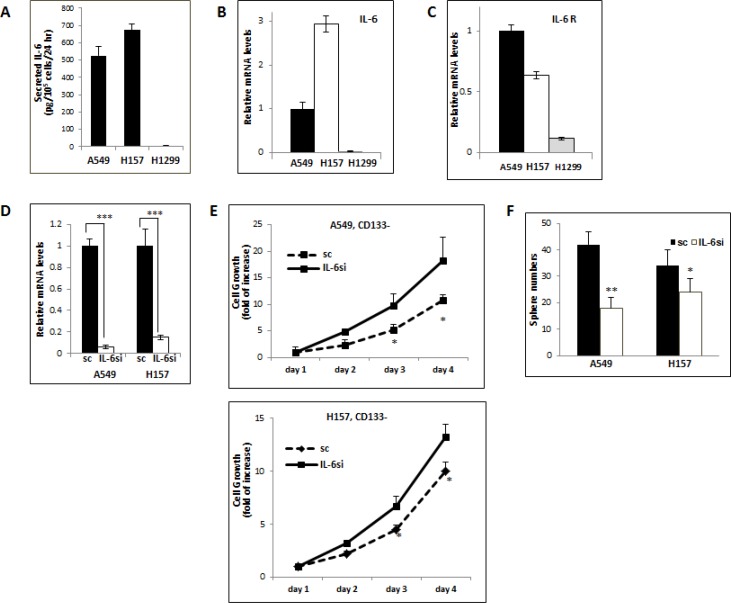
Opposite IL-6 effects on the growth/self-renewal of CD133− and CD133+ cells isolated from the IL-6 expression-manipulated A549 and H157 cell lines (**A**) IL-6 ELISA test. IL-6 secretions in supernatants of total A549, H1299, and H157 cells were analyzed by ELISA test. The IL-6 levels secreted by 10^5^ cells per 24 hours were presented. (**B** and **C**) qPCR results analyzing IL-6 and IL-6R levels in NSCLC cell lines. IL-6 (B) and IL-6R (C) expressions in 3 cell lines were analyzed by qPCR analyses. (**D**) qPCR analyses of IL-6 mRNA levels. The IL-6 mRNA expression in A549IL-6si/sc and H157IL-6si/sc cell sets were analyzed by qPCR tests. (**E**) Growth assay of CD133− cells obtained from A549IL-6si/A549sc and H157IL-6si/H157sc cell lines. (**F**) Sphere formation assay of CD133+ cells obtained from A549IL-6si/A549sc and H157IL-6si/H157sc cell lines. *p < 0.05, **p < 0.01, ***p < 0.001.

To further investigate the differential effects of IL-6 on the regulation of the growth/self-renewal of CD133– vs. CD133+ cells of NSCLC cell lines, we developed *in vitro* IL-6 expression-manipulated cell lines. To select appropriate cell lines for *in vitro* manipulation of IL-6 expression, we examined baseline IL-6 expression levels in the A549, H157, and H1299 NSCLC cell lines. The ELISA test results showed that A549 and H157 cell lines secreted IL-6 at high levels, but the H1299 cell line did not (Figure 3A). mRNA expression levels were consistent with the result of ELISA assay as shown in Figure 3B. The IL-6 receptor (IL-6R) level in the H1299 cell line was also found to be lower than the other two cell lines (Figure 3C).

Using the A549 and H157 cells that express high basal levels of IL-6, we knocked down the IL-6 levels via lentiviral transduction. The qPCR test results showed 90–95% IL-6 knockdown efficiency in these two cell lines (Figure 3D). We then isolated the CD133+ and CD133–cells from these IL-6 knocked down A549 (A549IL-6si) and H157 (H157IL-6si) cells as well as their scramble control (A549sc and H157sc) cells, and analyzed their growth. We found that growth rates of the CD133– cells of A549IL-6si and H157IL-6si cell lines were higher than those of scramble control cells (Figure 3E), suggesting an inhibitory role of IL-6 in CD133– cell growth. On the contrary, the self-renewal (sphere formation) of CD133+ population of the IL-6 knocked down cells was significantly reduced compared to their control cells as shown in the sphere formation assay (Figure 3F), supporting a promoter role of IL-6 in CD133+ cells' self-renewal. The contrasting results of IL-6 effects on the CD133– vs. CD133+ cells support dual effects of IL-6 in regulating the growth of CD133– (an inhibitory role) and CD133+ (a promoter role) cells of NSCLC cell lines.

### *In vivo* examination of the promoter role of IL-6 in CD133+ cells-derived tumor

To explore if our *in vitro* discovery of the promoter role of IL-6 in the self-renewal of CD133+ cells can be demonstrated *in vivo*, we performed investigations using human tumor xenografts in nude mice. We generated the *in vivo* model by subcutaneously inoculating CD133+ cells (1 × 10^4^), isolated from A549IL-6si (A549IL-6si-CD133+) vs. A549sc (A549sc-CD133+) cell lines into the bilateral flanks of mice and monitored tumor growth twice a week. We inoculated lower number of cells of CD133+ cells in consideration of the higher tumorigenicity of CD133+ cells than CD133– cells [27]. Indeed, when we inoculated the same number of CD133– cells (1 × 10^4^), we did not observe tumor generation (data not shown), supporting the higher tumorigenicity of CD133+ cells than CD133– cells.

**Figure 4:**
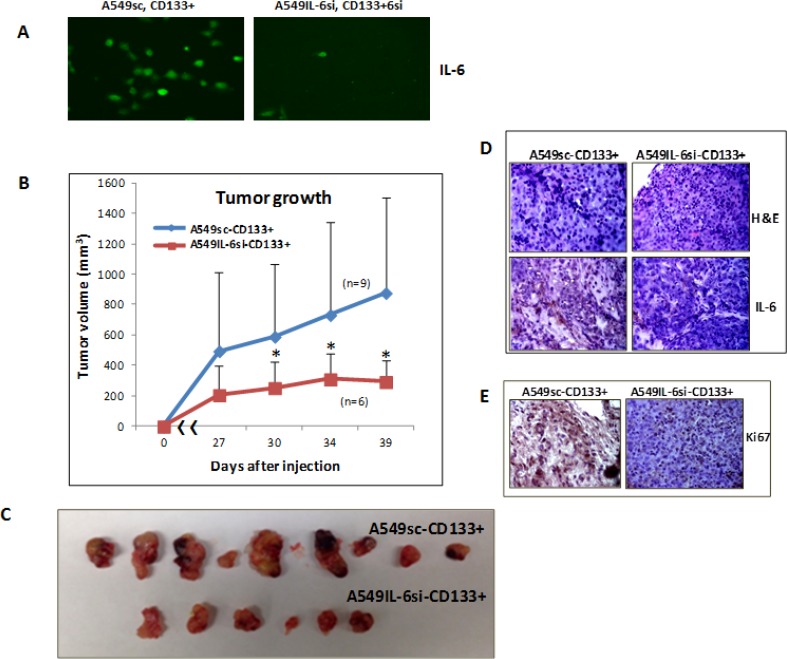
*In vivo* xenograft studies in mice (**A**) IF staining demonstrating IL-6 expression in CD133+ cells of A549IL-6si/sc cells used in the generation of human lung cancer xenografts. (**B**) Tumor growth in xenografts inoculated with A549IL-6si-CD133+ vs. A549sc-CD133+ cells (*n* = individual tumors in each group). (**C**) Tumors excised from the xenografts derived from CD133+ cells of A549IL-6si vs. A549sc cell lines. (**D**) H & E staining (upper) and IL-6 IHC staining (lower) of tumor tissues obtained from A549IL-6si-CD133+ and A549sc-CD133+ cells inoculated mice. (**E**) Ki67 IHC staining of tumor tissues obtained from the xenografts derived from the CD133+ cells of A549IL-6si/sc cell lines (*p < 0.05).

IL-6 expression in CD133+ cells from A549sc and A549IL-6si CD133+ cells were examined before inoculation into nude mice. Figure 4A shows immunofluorescence (IF) stains confirming IL-6 knockdown in the CD133+ cells isolated from the A549IL-6si cell line to be inoculated in mice. The growth of xenograft tumors from CD133+ cells derived from A549 sc vs. A549IL-6si cells were examined. As shown in Figure 4B, we found significantly retarded tumor growth in xenografts derived from CD133+ cells isolated from the A549IL-6si cell line compared to xenograft tumors derived from the CD133+ cells of the A549sc cell line, supporting that intrinsic IL-6 stimulated CD133+ cell growth in xenograft tumors. Tumors were subsequently excised from mice (Figure 4C) and H & E stain confirmed tumor phenotypes of IL-6 expression (Figure 4D). Immunohistochemical (IHC) staining with the proliferation marker Ki67 and the tumors derived from the CD133+ cells of A549IL-6si cell line showed significantly reduced Ki67 staining than the tumors derived from the CD133+ cells of the A549sc cell line (Figure 4E), again demonstrating reduced proliferation of CD133+ cells *in vivo* when IL-6 was knocked down. These *in vivo* results corroborated the *in vitro* finding that IL-6 played a positive role in promoting the CD133+ cell growth.

### Mechanistic investigation of signaling pathways modulated by IL-6 in CD133+ cells

**Figure 5:**
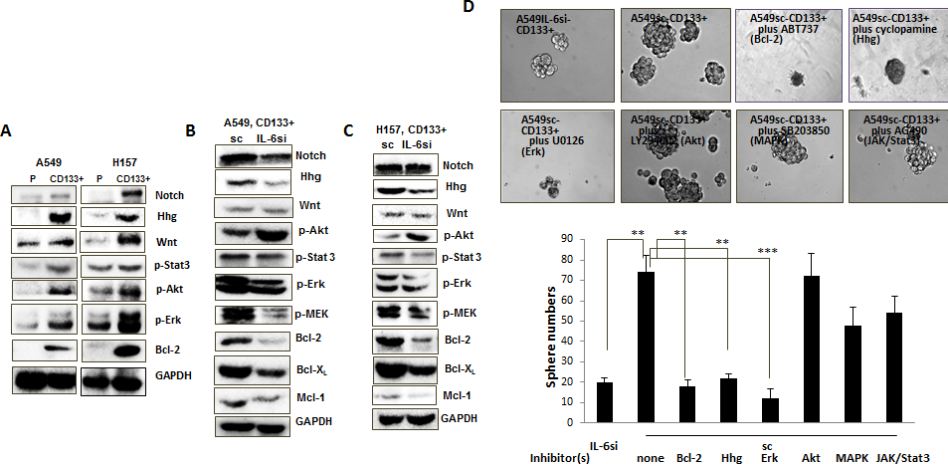
Mechanism dissection studies to reveal signaling pathways responsible for the role of IL-6 in promoting CD133+ cell growth (**A–C**) Western blot analyses using the cell extracts obtained from: A. Parental vs. CD133+ cells of A549 and H157 cell lines; B. CD133+ cells of A549IL-6si and A549sc cell lines; C. CD133+ cells of H157IL-6si and H157sc cell lines. (**D**) Inhibitor studies. A549IL-6si-CD133+ and A549sc-CD133+ cells were subjected into sphere formation assay, in the absence and presence of each inhibitor (ABT737, cyclopamine, U0126, LY294002, SB203850, and AG490) indicated. **p < 0.01, ***p < 0.001.

To explore the mechanism involved in the promoter role of IL-6 on CD133+ cells, we first examined the activation of several signaling pathways that had been reported to be important in CD133+ cells for self-renewal, including Sonic Hedgehog (Hhg) [31], Wnt [32], and Notch3 [33]. We detected the activation of these signaling pathways in CD133+ subpopulation for both A549 and H157 cells when compared with parental cells (Figure 5A). In addition to these signaling pathways, we also detected the activation of signaling pathways of Akt, extracellular signal-regulated kinases (Erk), Stat3, and mitogen activated protein kinase (MAPK)/ERK kinase (MEK) in CD133+ cells compared to the parental cells (Figure 5A). In addition, we found a marked increase of Bcl-2 (an anti-apoptotic marker) expression in CD133+ cells than parental cells (Figure 5A). We then investigated whether IL-6 signaling can further trigger the activation of these signaling pathways. Western blot analyses were performed in extracts of A549-IL-6si-CD133+ vs. A549sc-CD133+, and H157IL-6si-CD133+ vs. H157sc-CD133+ cells (Figure 5B: A549IL-6si-CD133+ vs. A549 sc CD133+; Figure 5C: H157IL-6si-CD133+ H157 CD133+ cells). The findings in both cell lines were consistent in that IL6-si CD133+ cells had lower levels of expression of Hhg, p-Stat3, p-Erk, p-MEK, Bcl-2, Bcl-xL, and Mcl-1expression when compared to respective control sc CD133+ cells of both cell lines. Data suggest that IL-6 expression in CD133+ cells modulates the up-regulation of Hhg, p-Stat3, p-Erk, p-MEK, Bcl-2, Bcl-xL, and Mcl but has no effect on Wnt, and also affects the down-regulation of Akt activation.

To further explore if the IL-6 expression-associated molecular signaling pathways discovered in Figure 5B and 5C may play a role in the growth and renewal of CD133+ cells, we applied available molecular inhibitors that specifically targeted these signaling pathways and investigated their effects on sphere formation of A549sc-CD133+ cells. Sphere formation assays were done in the presence of ABT737 (Bcl-2/Bcl-xL inhibitor) [34], cyclopamin (Hhg inhibitor) [35], U0126 (Erk/MEK inhibitor), LY294002 (Akt inhibitor), SB203850 (MAPK inhibitor), and AG490 (JAK/Stat3 inhibitor). We observed significantly reduced sphere numbers of A549sc-CD133+ cells by treatment with ABT737, cyclopamin, and U0126 (Figure 5D), and the level of reduction reached the similar level of sphere numbers of A549IL-6si-CD133+ cells. We did not observe any significant inhibition of self-renewal of A549sc-CD133+ cells treated with LY294002, SB203850, and AG490 (Figure 5D). Combined data from these inhibitor studies suggest that targeting the Bcl-2/Bcl-xL, Hhg, and Erk/MEK signaling pathways can potentially inhibit the IL-6-mediated CD133+ cell growth enhancement, providing a rationale of applying strategies by targeting these three signaling pathways, Erk/MEK, Bcl-2/Bcl-xL, and Hhg, in blocking the growth of CD133+ cells of NSCLC.

### Effects of IL-6 on the EMT/migration abilities of CD133– and CD133+ cells

**Figure 6:**
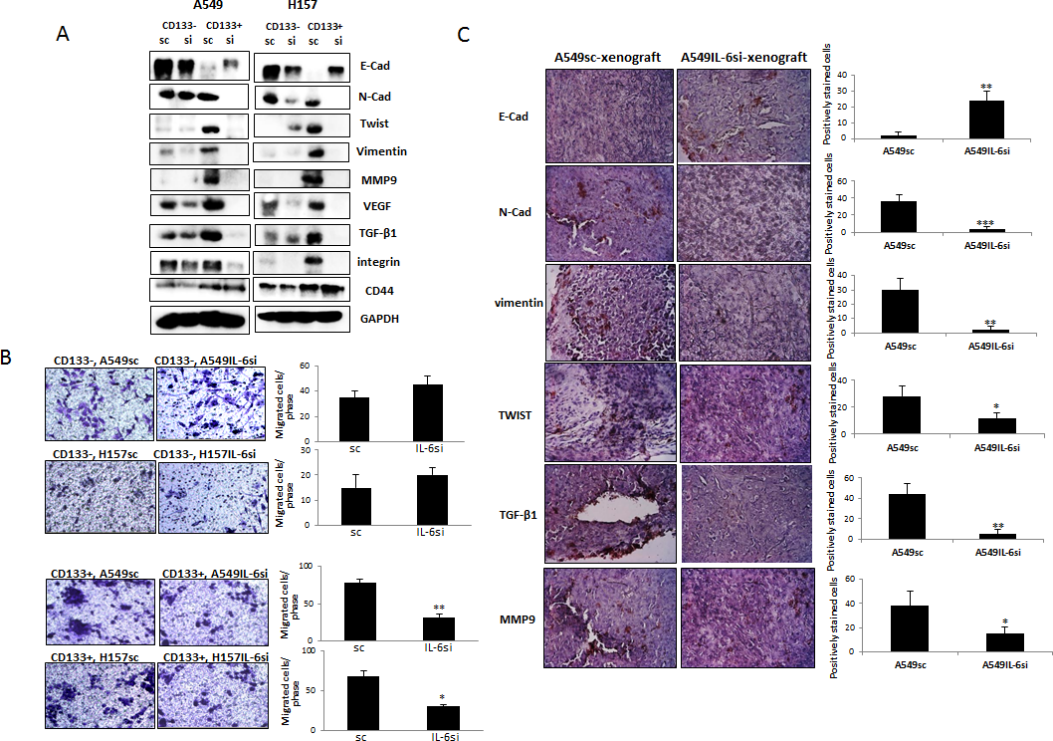
IL-6 regulation of EMT/metastasis in CD133+ cells of NSCLC (**A**) Western blot analyses of EMT/metastasis associated markers using the cell extracts obtained from CD133+ cells of A549IL-6si/sc and H157IL-6si/sc cell line sets. (**B**) Migration assay. CD133+ cells (1 × 10^4^) of A549IL-6si/sc and H157IL-6si/sc cell line sets were used in migration assay using transwell plates (8 μm pore). The migrated cells were stained with crystal blue and the positively stained cells were counted. Quantitation is shown on right. (**C**) IHC staining (EMT/metastasis associated markers) of tumor tissues obtained from the xenografts derived from the CD133+ cells of A549IL-6si/sc cell lines. Quantitation is shown on right. *p < 0.05, **p < 0.01, ***p < 0.001.

We next investigated whether IL-6 also differentially regulates the EMT process in CD133+ and CD133– cells. We isolated CD133– and CD133+ cells from A549IL-6si/sc and H157IL-6si/sc pairs, obtained cell extracts, and examined expression of the EMT (E-cad, N-cad, vimentin, and Twist) and metastasis-associated (MMP9, TGF-β1, VEGF) molecules in Western blot analyses. We found no significant regulation in expression of these molecules in CD133– cell sets, but observed significant IL-6 regulation in CD133+ cell sets (Figure 6A). We found significantly higher expression of these molecules in CD133+ cells of the IL-6 expressing A549sc/H157sc cell lines than the cells of IL-6 knocked down cell lines. The migration assay results support this finding. As shown in Figure 6B, no significant difference in migration ability of CD133– cells was observed whether or not they express IL-6 in cells, but significantly higher numbers of migrated cells were detected in the migration test using the CD133+ cells of sc cells compared to the cells of IL-6 knocked down cell lines. Differences in these markers were also observed in immunostaining of tumor tissues obtained in xenograft studies (Figure 4). When we stained tumor tissues of CD133+, A549IL-6si- and CD133+, A549sc-xenografts with the EMT/metastasis markers, we observed higher numbers of cells positively stained with these markers in tumor tissues of CD133+, A549sc-xenografts than those of CD133+, A549IL-6si-xenografts (Figure 6C). These results indicate that the IL-6 effect in promoting the EMT process in CD133+ cells and increasing their migration abilities were significant in CD133+ cells, not in CD133– cells.

We also investigated expression of several cell adhesion-associated molecules, such as CD44 and integrin. We could not detect the IL-6 regulation of CD44 in both population cell sets, but observed significant IL-6 regulation in integrin levels in CD133+ cell sets (Figure 6A). The integrin level was significantly higher in CD133+ cells of the IL-6 expressing A549sc/H157sc cells than in the cells of IL-6 knocked down cell lines. Consistently, we observed aggregation of CD133+ cells of the IL-6 expressing A549sc/H157sc cell lines in migration tests.

## DISCUSSION

In this report, we discovered differential roles of IL-6 in regulation of the growth/self-renewal of two subpopulation (CD133+ CSC-like, CD133–) cells of NSCLC cell lines. We found that IL-6 promoted the self-renewal of CD133+ cells, but inhibited the growth of CD133– cells. The IL-6 role in promoting growth of CD133+ cells has been implicated in previous reports [22, 23], but we revealed the dual roles of IL-6 in these two subpopulation cells, which clarifies the controversial issues on the IL-6 role in growth of total cells [15, 16, 24].

Our results with the exogenously added IL-6 on growth of CD133– cells were not consistent among these 3 cell lines. We detected almost no effect of IL-6 on the growth of CD133– cells of A549 and H157 cell lines that bear high basal levels of IL-6, while observing an inhibitory effect on the growth of CD133– cells of H1299 cell line that did not express IL-6. It is probable that demonstrating inhibitory effects in cell lines expressing high levels of IL-6 (A549 and H157) may be difficult. However, our further experimental approach of *in vitro* manipulation of intracellular IL-6 level enabled us to detect an inhibitory effect of IL-6 on CD133– cell growth (Figure 3E). This result suggests that the autocrine IL-6 signals may be more critical than the paracrine IL-6 signals in triggering the inhibitory effect.

Contrasting the CD133– cell studies results, our *in vitro* and *in vivo* data clearly showed the promoter role of IL-6 in regulating the CD133+ CSC-like cells growth. It may account for the important implication of IL-6 in lung cancer progression and chemo- and radioresistance. Mizutani et al. [36] showed that blocking IL-6 increased chemosensitivity to cisplatin in renal cancer cells, and our recently published results also showed that IL-6 plays a role in chemoresistance in NSCLC cells by increasing DNA repair and blocking apoptosis [37]. Moreover, our data using the CD133+ and CD133– cells of A549 and H157 cell lines showed that CD133+ cells have higher resistance than CD133– cells to cisplatin treatment (unpublished results), so we speculate that the role of IL-6 in chemoresistance may be more critical in CD133+ cells than in CD133– cells. In addition, recent studies in our laboratory revealed the importance of IL-6 in rendering radioresistance to CD133+ cells of lung cancer (manuscript *in press*). Therefore, although CD133+ cells comprise only a minority portion of cells in the parental cell lines (0.8–8.2% Figure 1A), we believe that the IL-6 role in promoting CD133+ cell growth may be important and have high clinical significance.

However, tumor tissues are thought to be hetero-geneous in IL-6 expression as the previous results on NSCLC cell lines indicated IL-6 expression in only 53% of NSCLC cell lines among the NSCLC cell lines tested [15, 16] and the report on patient tumor tissues also showed heterogeneous expression of IL-6 in lung tumor tissues [14]. In addition, the IL-6 role in the milieu of the tumor microenvironment is considered clinically important because there is a report indicating the interaction of IL-6 with other molecules in affecting these cells' growth [38]. Therefore, it will be necessary to isolate CD133+ and CD133– cells from tumor tissues and investigate the IL-6 role to understand the IL-6 effect on growth of CD133+ and CD133– cells.

We next showed that the IL-6 expressing CD133+ cells have significantly higher expression of EMT-related molecules (lower E-Cad, higher N-Cad, vimentin, and TWIST), as well as higher expression of metastasis-related molecules (MMP9, TGF-β1) than IL-6 knocked down CD133+ cells (Figure 6A). The IL-6 effect was slightly observed in CD133– cell sets, but the effect was not as significant as in CD133+ cell sets. These *in vitro* data were also proved in in tumor tissue analyses.

TGF-β1 is also suggested to be important in inducing the EMT process [39] and we observed higher expression of this molecule in the IL-6 expressing CD133+ cells than IL-6 knocked down CD133+ cells. VEGF has been known to be involved in angiogenesis process [40], but a recent publication showed that VEGF is important in migration of mesenchymal stem cells [41]. Therefore, we speculate that the IL-6-mediated high expression of VEGF may also contribute to the increased migration ability of CD133+ cells.

It is interesting to note that the IL-6 expressing CD133+ cells showed aggregation in the migration tests (Figure 6B). In investigating several adhesion-associated molecules, we found integrin is highly expressed in the IL-6 expressing CD133+ cells compared to the IL-6 knocked down CD133+ cells, but not much difference was detected in CD44 level. As the aggregated tumor cells were suggested to bear higher metastatic ability than non-aggregated cells [42], we believe the IL-6 regulation of aggregation of CD133+ cells may also have a clinical significance and further studies are thought to be necessary.

The anti-IL-6 strategy is expected to be effective on blocking growth and EMT/metastatic abilities of CD133+ cells. It has been suggested in lung cancer therapeutics and already been applied in clinical trials [21, 43]. However, using anti-IL-6 therapy to target the growth of CSC and CSC-derived EMT/metastasis has never been attempted.

In addition, given the complexity of the physiological activities of IL-6 in producing both pro- and anti-inflammatory effects in the immune system [44], any therapeutic approach using anti-IL-6 agents may result in complicated and unforeseen untoward outcomes. In this respect, selectively targeting IL-6 downstream signaling may be a better strategy than anti-IL-6 agents and this strategy may come from the result of our mechanistic investigation. In the mechanism dissection studies on revealing signaling pathways responsible for the IL-6 role in promoting CD133+ cells growth, we found several signaling pathways including Hhg, Wnt, and Notch that were up-regulated in CD133+ cells compared to total cells. We also found that IL-6 knockdown decreased the expression of Hhg, p-stat 3, p-Erk, p-MEK, Bcl-2, Bcl-xL, and Mcl-1, but not the Wnt and p-Akt signaling (Figure 5). Further inhibitor studies confirmed that Hhg, Bcl-2, and Erk/MEK are the essential signaling pathway or molecules in triggering the promoter role of IL-6 in CD133+ cell growth. Su et al. [45] earlier suggested that IL-6 modulated Hhg signaling in leukemia, but our finding of IL-6 modulation of Hhg signaling in CD133+ CSC-like cells of NSCLC has not been reported previously. Activation of Bcl-2 has been reported in prostate CSC studies [46], but its activation and the regulation by IL-6 signaling in lung cancer CSCs have not been addressed before. Taking together findings from our mechanism studies, our data suggest that Hhg, Bcl-2/Bcl-xL, and Erk/MEK are important target molecules in triggering the IL-6 effect on promoting CD133+ cell growth and expansion. In lung cancer therapeutics, a pilot trial of combined use of anti-Bcl-2 G3139 antisense oligonucleotide and paclitaxel has been applied [43], but no objective responses were observed. Likewise, the therapeutic approaches targeting Hhg pathway have also been proposed in several types of cancers, including lung cancer [47], but effects of therapies blocking these two signaling pathways to retard CSCs growth have never been tested in the clinical setting.

Nonetheless, we did not investigate possible regulation of IL-6 on other signaling pathways, such as IGF and NFκB, which were also reported to be important in CSC growth in other cancers in this study. On the other hand, the IL-6 regulation of epigenetic modification was also reported to be important in CSC growth [23]. We speculate that IL-6 can modulate several different ways to control CSC growth and through the signaling pathways we revealed in this study may be one of them.

To reveal target molecules in triggering the IL-6 effect in increasing the EMT/metastasis and aggregation of CD133+ cells, we may need further mechanism dissection studies. However, we speculate that integrin may be one of the IL-6 downstream molecules we may consider to target to block the CSC-mediated metastasis.

## MATERIALS AND METHODS

### Cell culture

A549, H1299, and H157 cell lines were purchased from the American Type Culture Collection (ATCC, Manassas, VA) and cultured in RPMI 1640 with 10% FBS. CD133+ CSCs were cultured in DMEM/F12 medium supplemented with ITS (insulin, transferin, selenium, Invitrogen), 20 ng/ml EGF (Invitrogen), and 20 ng/ml FGF (Invitrogen). All cells were maintained in a humidified 5% CO_2_ environment at 37°C.

For inhibition studies of sphere formation of CD133+ cells, we applied the inhibitors LY294002 (10 μM, Sigma, St Louis, MO), SB203850 (10 μM, Sigma, St Louis, MO), AG490 (5 μM, Cell Signaling, Danvers, MA), U0126 (10 μM, Cell Signaling, Danvers, MA), ABT737 (0.1 μM, Selleckchem Houston, TX), and cyclopamine (5 μM, Selleckchem Houston, TX) that inhibit Akt, MAPK, and JAK/Stat3, Erk/MEK, Bcl-2, and Hhg signaling pathways, respectively.

### Isolation of CD133+ CSC-like cells using microbead immunoseparation

Cells (2 × 10^7^) were detached from tissue culture plates with 5 mM EDTA, centrifuged, and incubated with magnetic microbeads conjugated with anti-CD133 antibody (Miltenyi Biotec, Cambridge, MA). The bead-bound cells (CD133+) and unbound cells (CD133–) were separated in QuadroMACS^™^ Separation Unit (Miltenyi Biotec, Cambridge, MA). The purity of the isolated CD133+ cells was confirmed by flow cytometric analyses, and by Western blot analyses. The isolated CD133+ cells were cultured in stem cell media as described above.

### Plasmids and cell infection using lentiviral system

For the incorporation of IL-6 siRNA or scrambled control plasmids into A549 and H157 cells, lentiviral plasmids carrying either control (scramble) or IL-6 siRNA (pLenti-II vector) (Applied Biological Materials Inc, Canada) sequence were transfected into 293T cells as a mixture of pLent-II-IL-6 siRNA, psPAX2 (virus-packaging plasmid), and pMD2G (envelope plasmid) (4:3:2 ratio) using PolyFect Transfection reagent (Qiagen, Valencia, CA). The virus supernatants were infected into A549 and H157 cells and the positive cell clones were selected by puromycin (2 μg/ml) (Sigma) and then maintained in media containing 0.1 μg/ml puromycin.

### Growth assay and sphere formation assay

CD133– cells or total cells of A549, H1299, and H157 cell lines were seeded into 24-well plates (1 × 10^4^ cells/well) and cell growth at different time points (days 2, 4, and 6) was analyzed by cell counting. For sphere formation assay, single-cell suspensions (1 × 10^3^ cells) were mixed with cold Matrigel (BD, Franklin Lakes) (1:1 ratio, v/v, total volume of 100 μl)) and the mixture was placed along the rim of the 24-well plates. The culture plates were placed in 37°C incubator for 10 min to let the mixture solidify and 500 μl medium was then added into the wells. In testing inhibitor effects, appropriate concentration of individual inhibitor was added into the medium. Sphere numbers of higher than 50 μm in diameter were counted after 7–14 days under an Olympus light microscope. A minimum of three triplicate experiments were performed.

### IL-6 ELISA

IL-6 in the supernatant of A549, H157, H1299, A549sc, A549IL-6si, H157sc, and H157IL-6si cell lines was determined by the ELISA kit according to the manufacturer's instructions (BD, Franklin Lakes). The secreted IL-6 level was normalized by cell number.

### RNA extraction and quantitative PCR (qPCR) analysis

Total RNAs were isolated using Trizol reagent (Invitrogen). One μg of total RNA was subjected to reverse transcription using Superscript III transcriptase (Invitrogen). qPCR was conducted using a Bio-Rad CFX96 system with SYBR green to determine the mRNA expression level of a gene of interest. Expression levels were normalized to GAPDH level.

### Western blot analysis

Cells were lysed in RIPA buffer (50 mM Tris-Cl at pH 7.5, 150 mM NaCl, 1% NP-40, 0.5% sodium deoxycholate, 1 mM EDTA, 1 μg/mL leupeptin, 1 μg/mL aprotinin, 0.2 mM PMSF) and proteins (20–40 μg) were separated on 8–10% SDS/PAGE gel and then transferred onto PVDF membranes (Millipore, Billerica, MA). After the blocking procedure, membranes were incubated with primary antibodies, HRP-conjugated secondary antibodies, and visualized in Imager (Bio-Rad) using ECL system (Thermo Fisher Scientific, Rochester, NY). GAPDH, bcl-2, bcl-xL, Notch, Hhg, Wnt1, CD44, and IL-6R antibodies were from Santa Cruz Biotechnology (Santa Cruz, CA). CD133 antibody was from Miltenyi Biotec (San Diego, CA), and ALDH antibody was obtained from BD Biosciences (San Jose, CA). p-Stat3, p-Akt, p-Erk, p-MEK, Oct4, Nanog, Sox2, and Mcl-1 antibodies were purchased from Cell Signaling (Danvers, MA). The antibodies of E-cad, N-cad, Twist, MMP9, and TGF-β1 were obtained from Abgent (San Diego, CA) and Integrin and VEGF antibodies were purchased from Abcam (Cambridge, UK).

### Migration assay

Cells (CD133– and CD133+ cells isolated from A549IL-6si/sc and H157IL-6si/sc pairs, 1 × 10^4^) were placed in upper layer of transwell plates (Corning, 8 μm pore size, 24 well plates, no serum containing media). Migrated cells at the end of 24 hours of incubation were visualized by staining with crystal blue solution and counted under microscope. Three independent experiments (with triplicates) were done and quantitation was obtained from average numbers of positively stained cells in 3 phases.

### *In vivo* xenograft studies

The CD133+ cells (1 × 10^4^ cells per site) that had been isolated from A549sc (control group) and A549IL-6si (test group) cell lines were subcutaneously injected (100 μl total injection volume, mixture of 1:1, media: Matrigel, v/v) into 8-week old female nude mice (NCI) (10 mice per group for a total of 20 mice). Tumor development and growth were monitored twice a week.

### Histology and immunohistochemistry

Tumor tissues obtained were fixed in 10% (v/v) formaldehyde in PBS, embedded in paraffin, and cut into 5-μm sections. Tumor tissue sections were deparaffinized in xylene solution and rehydrated, and immunostaining was performed. Similar antibodies used in Western blot analysis were applied in staining procedure, and the Ki67 antibody was from Abcam (Cambridge, MA). For Ki67 staining, the antigen retrieval process was performed in 10 mM Citric buffer, pH 6.0 for 20 minutes using a cooker prior to staining. After staining, tissues were counterstained by Hematoxylin.

### Statistics

The data values were presented as the mean ± SEM. Differences in mean values between two groups were analyzed by two-tailed Student's *t* test. *p* ≤ 0.05 was considered statistically significant.
